# Single-polyp metabolomics for coral health assessment

**DOI:** 10.1038/s41598-024-53294-8

**Published:** 2024-03-05

**Authors:** Akira Iguchi, Mariko Iijima, Nanami Mizusawa, Yoshikazu Ohno, Ko Yasumoto, Atsushi Suzuki, Shunichi Suga, Ken Tanaka, Kei Zaitsu

**Affiliations:** 1 Geological Survey of Japan, National Institute of Advanced Industrial Science and Technology (AIST), 1-1-1 Higashi, Tsukuba, Ibaraki 305-8567 Japan; 2 Research Laboratory on Environmentally-Conscious Developments and Technologies [E-Code], National Institute of Advanced Industrial Science and Technology (AIST), Tsukuba, 305-8567 Japan; 3 School of Marine Biosciences, Kitasato University, 1-15-1 Kitasato, Minami, Sagamihara, Kanagawa 252-0373 Japan; 4 Research Laboratories, KOSÉ Corporation, 48-18, Sakae-cho, Kita-ku, Tokyo, 114-0005 Japan; 5 Multimodal Informatics and Wide-Data Analytics Laboratory (MiWA-Lab.), Faculty of Biology-Oriented Science and Technology, Kindai University, Nishimitani, Kinokawa, Wakayama, 649-6493 Japan

**Keywords:** Environmental impact, Ecophysiology

## Abstract

Coral reef ecosystems supported by environmentally sensitive reef-building corals face serious threats from human activities. Our understanding of these reef threats is hampered by the lack of sufficiently sensitive coral environmental impact assessment systems. In this study, we established a platform for metabolomic analysis at the single-coral-polyp level using state-of-the-art mass spectrometry (probe electrospray ionization/tandem mass spectrometry; PESI/MS/MS) capable of fine-scale analysis. We analyzed the impact of the organic UV filter, benzophenone (BP), which has a negative impact on corals. We also analyzed ammonium and nitrate samples, which affect the environmental sensitivity of coral-zooxanthella (Symbiodiniaceae) holobionts, to provide new insights into coral biology with a focus on metabolites. The method established in this study breaks new ground by combining PESI/MS/MS with a technique for coral polyps that can control the presence or absence of zooxanthellae in corals, enabling functions of zooxanthellae to be assessed on a polyp-by-polyp basis for the first time. This system will clarify biological mechanisms of corals and will become an important model system for environmental impact assessment using marine organisms.

## Introduction

Currently, there is great concern about adverse effects on ecosystems due to discharge of anthropogenic chemicals into the environment and disruption of nutrient cycles. In terms of the planetary boundary framework demarcating a global safe operating space for humanity, six of the nine boundaries have already been transgressed, including not only climate change and ocean acidification, but also discharge of anthropogenic chemicals and disruption of nutrient cycles^1^, and there is a growing awareness of the necessity to regulate chemicals in the ocean, including microplastics. Against this backdrop, there is a growing need for assessment systems to determine how chemicals adversely affect marine organisms. Tropical coral reef ecosystems provide a range of important ecosystem services to human society, including coastal protection, fisheries, and tourism^2,3^, but a global decline of reef-building corals is occurring because of coral sensitivity to environmental change at global and regional scales^4–7^. Corals may also be adversely affected by anthropogenic chemicals^8–10^. Considering that corals are keystone organisms of marine ecosystem, establishment of a reliable environmental impact assessment system using corals is urgently needed.

In recent years, sunscreen products and their ingredients have also been reported to adversely affect corals^10–18^. The organic UV filter, benzophenone (BP or oxybenzone), harms corals in laboratory studies^11,12,18^, and bans have been issued in some areas^19–21^. However, previous studies assessing effects of sunscreens on corals continue to be controversial due to lack of analytical verification of exposure concentrations, poor controls, and lack of environmental relevance^12^. Moreover, establishment of standard toxicological assessment protocols is still wanting.

**Figure 1:**
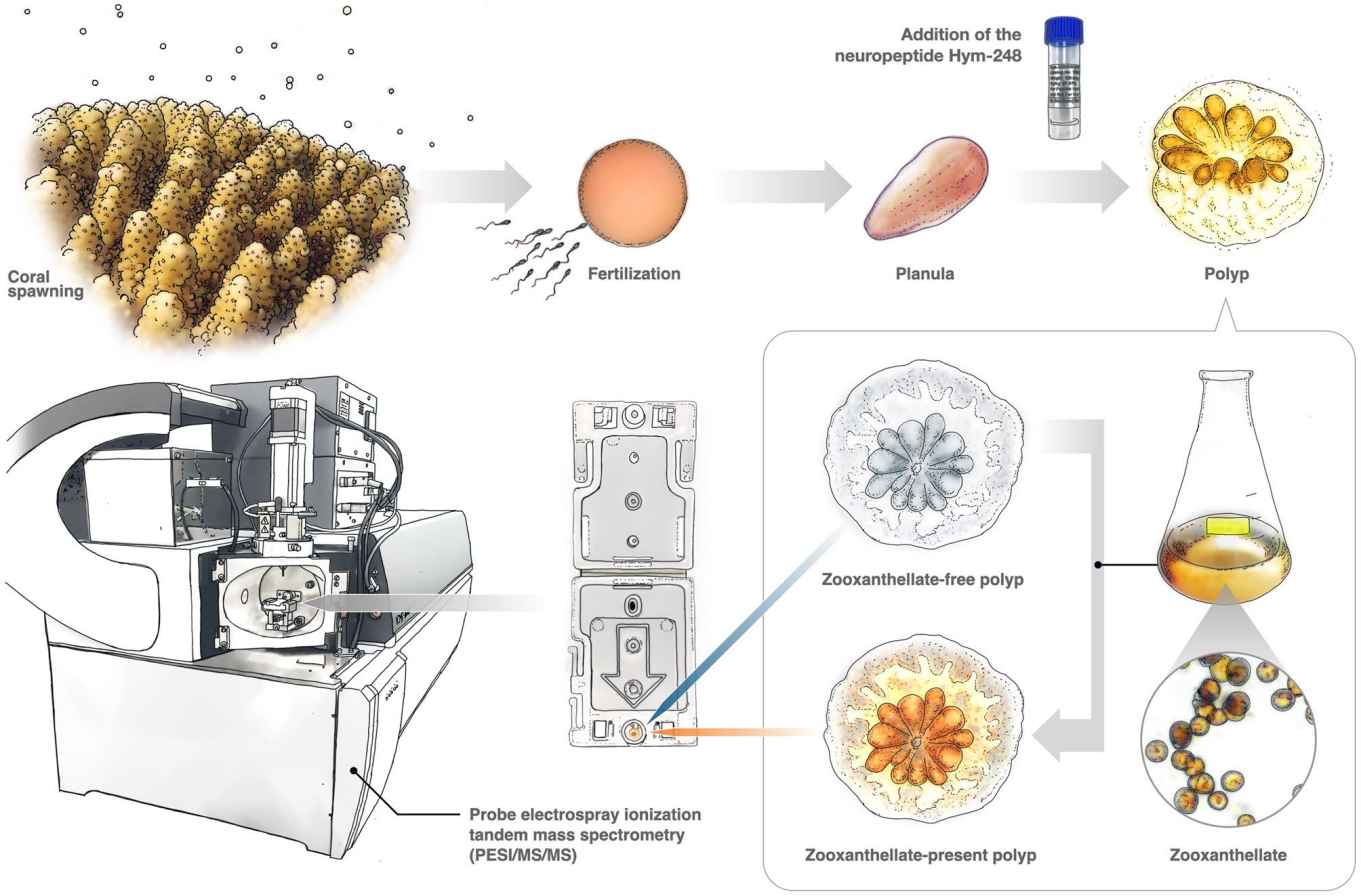
Schematic of the experimental flow using coral polyps and probe electrospray ionization/tandem mass spectrometry (PESI/MS/MS). Holobionts can be created by artificially adding zooxanthellate. Metabolomic data can be acquired within 2.4 min by fixing the sample plate under a fine needle that acquires metabolite data.

Previous studies assessing chemical effects on corals have used mainly mature coral fragments, but rearing experiments using coral fragments are difficult without a flowing seawater system, and it is also difficult to ensure sufficient space for rearing an adequate number of replicates. Coral primary polyps obtained during simultaneous spawning are smaller than coral fragments (Fig. 1), so they can be reared in petri dishes. Necessary numbers of replicates can easily be achieved and chemical exposures can be controlled^8,22^. Rearing of healthy coral primary polyps is an essential step in coral larval recruitment, and assessment of impacts during the primary polyp stage is extremely important from the perspective of maintaining coral populations. This life history stage is considered more sensitive to environmental changes than mature coral colonies (reviewed in^23^), making them excellent for sensitive toxicological assessments. Another advantage is that coral primary polyps produced from *Acropora* species can be artificially infected with zooxanthellae (Symbiodiniaceae) to carry out response assessments in the presence or absence of zooxanthellae, as larvae of *Acropora* species are zooxanthella-free^24^. As corals maintain a symbiotic life history with zooxanthellae, environmental responses also need to be assessed in coral holobionts. The state of this coral-zooxanthellae symbiosis varies in sensitivity to high water temperature stress depending on surrounding nutrient concentrations (ammonium and nitrate as nitrogen sources)^25^. However, it is unclear how metabolite levels that respond to changes in seawater nutrient concentrations are altered by zooxanthellae.

Biological parameters such as survivorship, growth rate (calcification rate) and tentacle movement are used to assess coral health in coral rearing experiments^8,22,24,26^, but there are limitations to assessing health based on morphological and behavioral observations. Metabolomic analysis, which is commonly used in medicine and other fields, allows numerous metabolites to be quantified simultaneously, making it possible to extract metabolites of interest according to the target life phenomenon. In addition, metabolomic analysis can also be interpreted as phenotypic analysis and is therefore excellent for detecting potential effects that are not discoverable by other methods, making it suitable for environmental impact and stress assessment. Conventional metabolomic analysis requires a relatively large amount of sample, whereas probe electrospray ionization/tandem mass spectrometry (PESI/MS/MS), a newly developed technique in medicine, enables local analysis with an ultra-fine metal needle (tip diameter: 700 nm) without sample pre-treatment. It has been applied to fine-scale analysis of mouse liver^27^, mouse brain^28,29^ and agricultural crops^30^. Considering the above background, if metabolomic analysis can be performed on a single coral primary polyp by PESI/MS/MS, it would provide a powerful new platform for simple and rapid assessment of exposure to various chemical substances. We have therefore developed a new method for metabolomic analysis of single coral primary polyps of a reef-building coral, *Acropora* sp., to assess the impact of chemical exposure.

## Results and discussion

We used coral primary polyps produced from gametes obtained during coral spawning, and artificially added zooxanthellae to create primary polyps with and without zooxanthellae (Figs. 1 and S1), and conducted exposure experiments using BP, which has adverse effects on corals. Metabolite information was obtained for seven zooxanthella-free polyps in the control group, four BP-exposed polyps (Table S1), and for four holobionts in the control and six experimental groups (Table S2). For some samples, polyps were not punctured successfully with the needle and data could not be obtained. As for experiments involving nitrate or ammonium exposure, metabolite information was obtained for five polyps in the control, five in the nitrate-exposed and five in the ammonium-exposed zooxanthella-free polyps (Table S3), and for three polyps in the control, four in the nitrate-exposed and five in the ammonium-exposed polyps with zooxanthella-present polyps (Table S4). The variation in the number of data acquired may be due to the somewhat larger size of the holes in the plate (Fig. S1) compared to the polyps, which may have biased the position, and therefore requires smaller hole size in the future.

**Figure 2:**
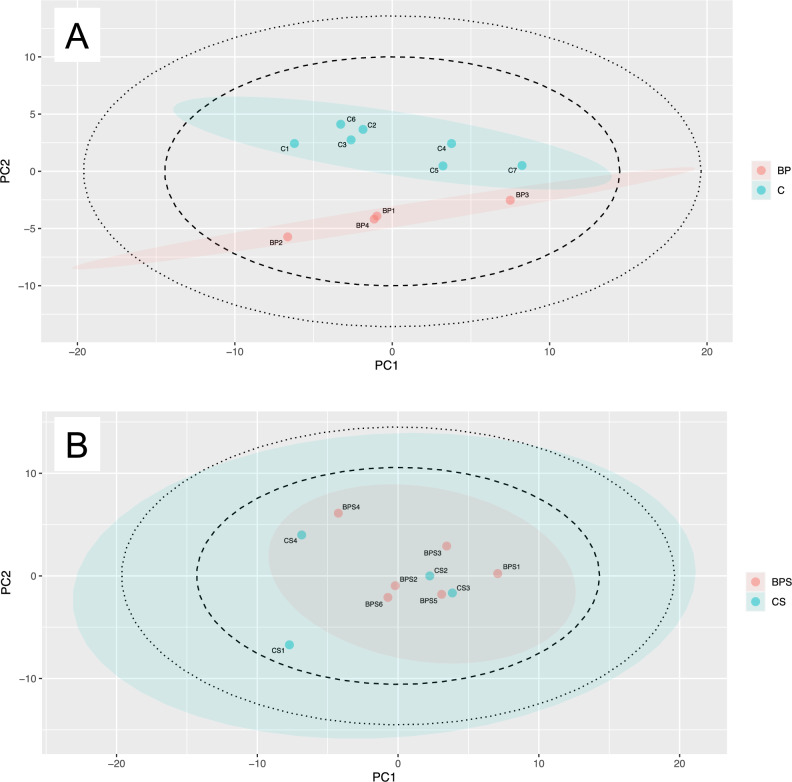
Principal component analysis (PCA) of the BP treatment. Dotted and solid circles in score plots indicate 95% and 99% confidence intervals for all plots, respectively. Colored circles indicate 95% confidence intervals for each group; (**A**) primary polyps without zooxanthellae; (**B**) primary polyps with zooxanthellae. PC1, first principal component; PC2, second principal component; BP, BP treatment, C, control and S, zooxanthellae present.

Using multivariate metabolite data, PCA readily segregated zooxanthella-free polyps with and without BP exposure (Fig. 2A), with nine metabolites showing significant differences between treatments (Fig. S2). On the other hand, zooxanthellate polyps exposed and unexposed to BP were not segregated (Fig. 2B) and no metabolites showed significant differences between treatments. In a previous study, toxicity of BP to cnidarians differed in the presence and absence of zooxanthellae^11^, and this was replicated in the present experiments. A previous study^11^ showed that symbiotic algae mitigate effects on host cnidarians by sequestering the phototoxic oxybenzone metabolite. In other words, symbiotic algae may prevent adverse effects of reactive oxygen species on corals caused by oxybenzone metabolites. In our metabolomic analysis, we found a reduction of some amino acids in zooxanthella-free-polyps under BP treatment (Fig. S2), and a previous study also found a reduction of amino acids such as glutamate under oxidative stress caused by high-water-temperature exposure^31^, suggesting suppression of metabolism due to oxidative stress. In addition, metabolomic analyses of coral species that inhabit relatively high-stress areas also show a trend toward low levels of arginine and other amino acids^32^. Reduced glutathione, an important intracellular antioxidant, is oxidized to eliminate reactive oxygen species^33,34^, and the decrease of reduced glutathione in BP treatment is a result of the reaction with ROS produced by phototoxicity of oxybenzone metabolites.

**Figure 3:**
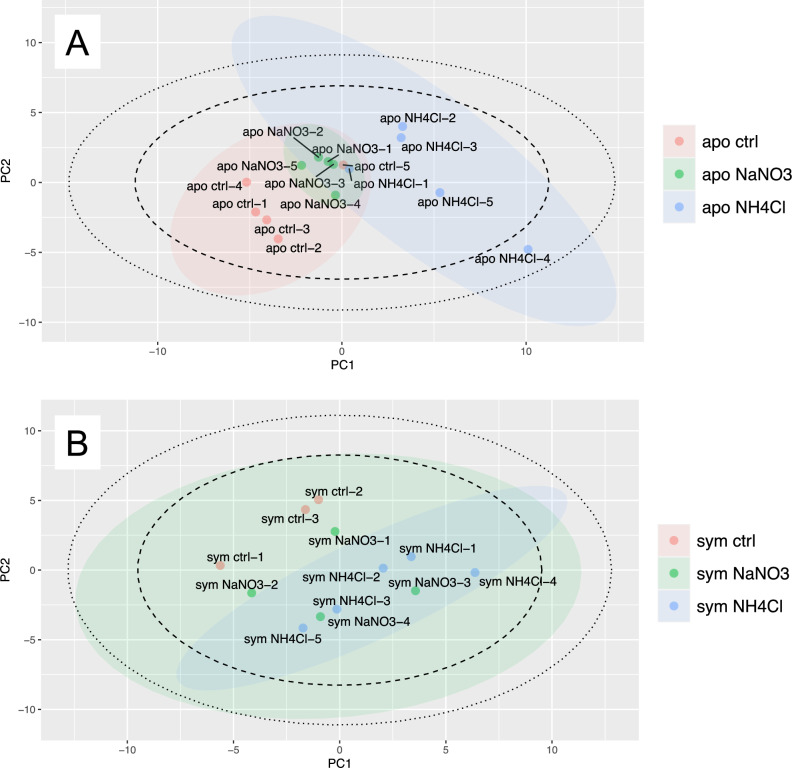
Principal component analysis (PCA) of nitrate and ammonium treatments. Dotted and solid circles in score plots indicate 95% and 99% confidence intervals for all plots, respectively. Colored circles indicate 95% confidence intervals for each group; (**A**) primary polyps without zooxanthellae; (**B**) primary polyps with zooxanthellae. PC1, first principal component; PC2, second principal component; apo, sym, without and with zooxanthellae, respectively; NaNO_3_, nitrate treatment; NH_4_Cl, ammonium treatment; ctrl, control.

PCA using multivariate data on metabolites obtained in nitrate and ammonium experiments confirmed group segregation in zooxanthella-free polyps with and without ammonium exposure (Fig. 3A). Among zooxanthella-free polyps, significant differences were observed in 13 metabolites between control and ammonium-exposure groups. Ten of these corresponded to amino acids and increased in the treatment group. In contrast, 3 of them corresponded to fatty acids and decreased in the treatment group (Fig. S3). On the other hand, in polyps with zooxanthellae, no group segregation was observed upon nitrate or ammonium exposure (Fig. 3B), and the only metabolite that showed significant differences in the treatment group was asparagine (Fig. S4). Previous studies have reported that enrichment of nitrate and ammonium is associated with effects on the energetic and redox status of corals^35^. In corals, nitrate enrichment induces oxidative stress and ammonium enrichment promotes amino acid synthesis and protein turnover^35^. Indeed, our experimental results showed a significant increase in amino acids such as lysine and valine upon ammonium exposure (Fig. S3). The decrease in fatty acids may have been due to their consumption for energy, as a result of accelerated amino acid synthesis caused by ammonium. Indeed, it has been reported that all three fatty acids that were reduced in the present study are abundant in corals and may be used for cell structure and energy storage^36,37^. Nitrate exposure did not change metabolite profiles associated with oxidative stress, but these differences from previous studies may reflect the different coral species and coral fragments used in those studies compared to this study^38^. Therefore, we recommend that PESI/MS/MS be applied to the mature corals and comparisons with the results obtained in the present study should be made. Recent studies also highlighted limitations of analyzing metabolites in holobionts^39^, emphasizing the need for analysis in independent component partners. With regard to symbiotic systems between cnidarians and zooxanthellae, experimental systems are well established in the anemone *Aiptasia*^40,41^, which is an important future target for PESI/MS/MS.

The platform established in this study, which combines a coral primary polyp experimental system with PESI/MS/MS, succeeded in comprehensively capturing metabolite changes in single coral polyps. Furthermore, by comparing responses in the presence and absence of zooxanthellae, it was possible to verify at the metabolite level whether effects of substance exposure occur in the coral itself or in the holobiont. One disadvantage of using coral primary polyps is that gametes can only be collected when a limited number of coral taxa spawn simultaneously, but *Acropora* sp. 1 used in this study, spawns approximately two months later than most *Acropora* species^42^, so we were able to observe coral mass spawning twice, allowing us to carry out multiple experiments. Although sample sizes were somewhat limited in this study, the method is scalable, as it is possible to assay other metabolites as well. The platform has the potential to be used not only for chemical exposure, but also to investigate other biological phenomena, such as responses of coral holobionts to high seawater temperatures and acidified seawater, tissue regeneration, and calcification of corals. It can potentially become an important model system to assess effects of environmental changes on marine organisms. This assessment system could be employed not only for the risk assessment of chemicals and other substances but also for evaluation of positive impacts, including enhanced growth and promotion of metabolic processes.

## Methods

### Material

Gametes of *Acropora* sp. 1^42^ inhabiting reef edges in the Ryukyu Islands were collected during mass spawning in 2020 and 2022. The number of coral colonies used was four in 2020 and six in 2022. Coral colonies used in this study were collected with permission from Okinawa Prefecture (permit numbers: 31–68 and 4–20). Metamorphic larvae were prepared from these gametes at 25 °C using 10 μM Hym-248 (Eurofins Genomics KK, Tokyo, Japan)^43^. Coral primary polyps were settled on glass-based dishes (No. 1S, thickness: 0.15–0.18 mm; IWAKI Glass, Tokyo, Japan) and submerged in oligotrophic natural seawater in a plastic box. Preparation of coral primary polyps followed our previous study^22^. Infection with zooxanthellae (Clade A; *Symbiodinium* sp.^44^) also followed our previous studies^24,45^ and infection status was visually confirmed.

To assess the effect of nitrogen loading, filtered seawater adjusted with 10 μM NH_4_Cl and 10 μM NaNO_3_ was prepared, and zooxanthella-infected and uninfected coral primary polyps (2020 gametes) were reared for 21 days. To assess effects of BP, we purchased 2-Hydroxy-4-methoxybenzophenone (equivalent to BP-3) from Tokyo Chemical Industry Co., Ltd. A vial containing a concentration of 10,000 mg/L of BP-3 in 2 mL of Dimethyl sulfoxide (DMSO) solution was placed directly on top of the lamp portion of a SLUV-4 UV lamp (ASONE, Osaka, Japan). This assembly was then enclosed in a cardboard box and irradiated with ultraviolet light at 254 nm for 24 h. Following irradiation, the solution was diluted with seawater to a final concentration of 2 mg/L, consistent with a prior study^11^, and used to culture both zooxanthella-infected and uninfected coral polyps (spawned in 2022) over 5 days. After the exposure experiment, coral primary polyps were removed from the glass plates with a knife, transferred prematurely to plates dedicated for PESI/MS/MS analysis and stored at -80 °C.

### PESI/MS/MS and statistics: PiTMaP platform

To perform metabolomic analysis of single coral polyps, we used probe electrospray ionization/tandem mass spectrometry (PESI/MS/MS) with an ultra-fine metal needle (tip diameter: 700 nm). Regarding data analysis, the PiTMaP platform^29^ was used, incorporating automated analysis from data collection by the mass spectrometer through a data pipeline using the statistical software package, R. Metabolome analysis with PiTMaP was carried out using a PESI source with an LCMS-8040 tandem mass spectrometer (Shimadzu, Kyoto, Japan). The measurement mode of the mass spectrometer was set to the scheduled-selected reaction monitoring (SRM) method to optimize conditions for multi-component detection. Ionization was performed in PESI-positive and -negative modes. The 72 components targeted included metabolites of the glycolytic, citric acid, urea, pentose phosphate, glutathione, and methionine pathways, allowing assessment of metabolites related to oxidative stress in addition to central metabolism. Acquisition time and the PESI probe cycle were optimized in accordance with our previous report^29^, and metabolomic data were acquired within 2.4 min per sample. Coral primary polyps were frozen samples, thawed on ice and placed on dedicated sample plates for PiTMaP. Just prior to analysis, pseudo-homogenization was performed by high-speed puncture using a PESI probe needle (100 times; Movie S1).

In accordance with our previous study^29^, the PiTMaP data pipeline with R software (version 3.6.3) was used for multivariate analysis (principal component analysis) and projection to latent structure-discriminant analysis (PLS-DA) and tests for differences in means between treatments were performed for metabolites refined by PLS-DA (VIP value > 1.0). To control for multiple comparisons of metabolomic data, p-values obtained from Tukey's or Welch’s tests were adjusted with the false discovery rate (FDR) procedure of Benjamini and Hochberg and adjusted p-values were reported as q-values. The PiTMaP data pipeline can complete such analyses and outputting figures and tables within 1 min.

### Supplementary Information

Supplementary Legends.

Supplementary Figure 1.

Supplementary Figure 2.

Supplementary Figure 3.

Supplementary Figure 4.

Supplementary Tables.

Supplementary Video 1.

## Data Availability

Data used in this study are summarized in the Supplementary tables.

## Code availability

Codes used in this study are those used in the previous study^29^.
