# Supplementary material for: Single-polyp metabolomics for coral health assessment
Source: Sci Rep. 2024 Mar 5;14:3369. doi: 10.1038/s41598-024-53294-8 (PMC10914721; doi:10.1038/s41598-024-53294-8)
Supplement: Supplementary file 1 — Supplementary Legends. [file 41598_2024_53294_MOESM1_ESM.docx]

**Supplementary Figure Legends**

Figure S1. Schematic of the experimental flow using coral polyps and probe electrospray ionization/tandem mass spectrometry (PESI/MS/MS). Holobionts can be created by artificially adding zooxanthellate. Metabolomic data can be acquired within 2.4 min by fixing the sample plate under a fine needle that acquires metabolite data.

Figure S2. Box-and-whisker plot of metabolites significantly altered in BP-treated zooxanthellae-free primary polyps between control and treatment groups. In zooxanthella-free polyps, nine metabolites showed significant differences between treatments.

Figure S3. Box-and-whisker plot of metabolites significantly altered between control and treatment in ammonium-treated zooxanthella-free primary polyps. In zooxanthella-free polyps, significant differences were found in 13 metabolites between control and ammonium treatments, 10 of which corresponded to amino acids and were increased in the treatment, and three fatty acids, which decreased in the treatment.

Figure S4. Box-and-whisker plot of metabolites significantly altered in ammonium-treated zooxanthellate polyps between control and treatment. In holobionts, the only metabolite that showed significant differences between treatments was asparagine.

Movie S1. Video of coral polyps being processed by PESI/MS/MS. Metabolomic data can be acquired by fixing the sample plate under a fine needle that acquires metabolite data within 2.4 min.

Table S1 Raw data of BP treatment using primary polyps without zooxanthellae.

Table S2 Raw data of BP treatment using primary polyps with zooxanthellae.

Table S3 Raw data of nitrate and ammonium treatments using primary polyps without zooxanthellae.

Table S4 Raw data of nitrate and ammonium treatments using primary polyps with zooxanthellae.
