# Supplementary figures and images for: Single-polyp metabolomics for coral health assessment
Source: Sci Rep. 2024 Mar 5;14:3369. doi: 10.1038/s41598-024-53294-8 (PMC10914721; doi:10.1038/s41598-024-53294-8)

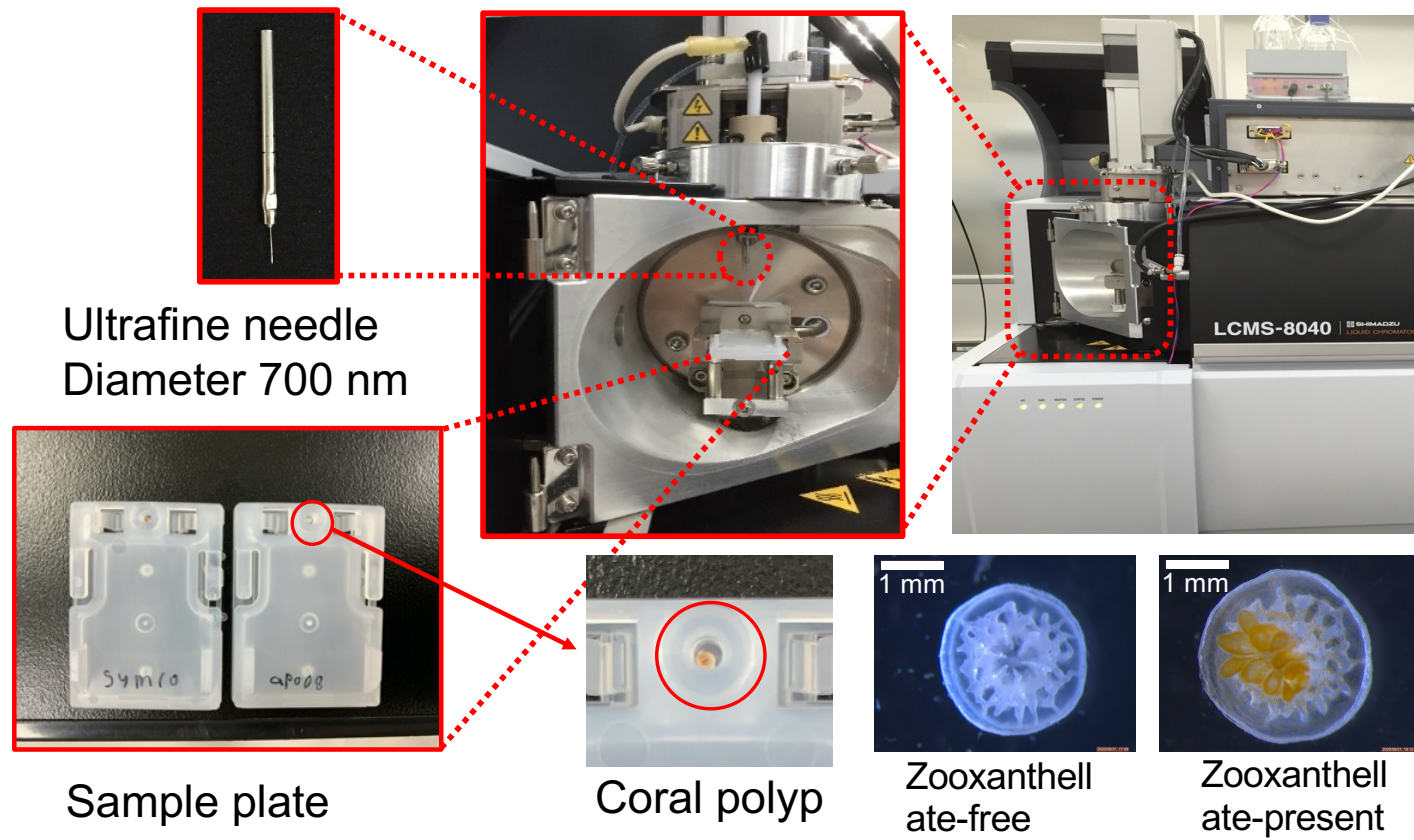

Figure S1

Supplement: Supplementary file 2 — Supplementary Figure 1. [file 41598_2024_53294_MOESM2_ESM.pdf]

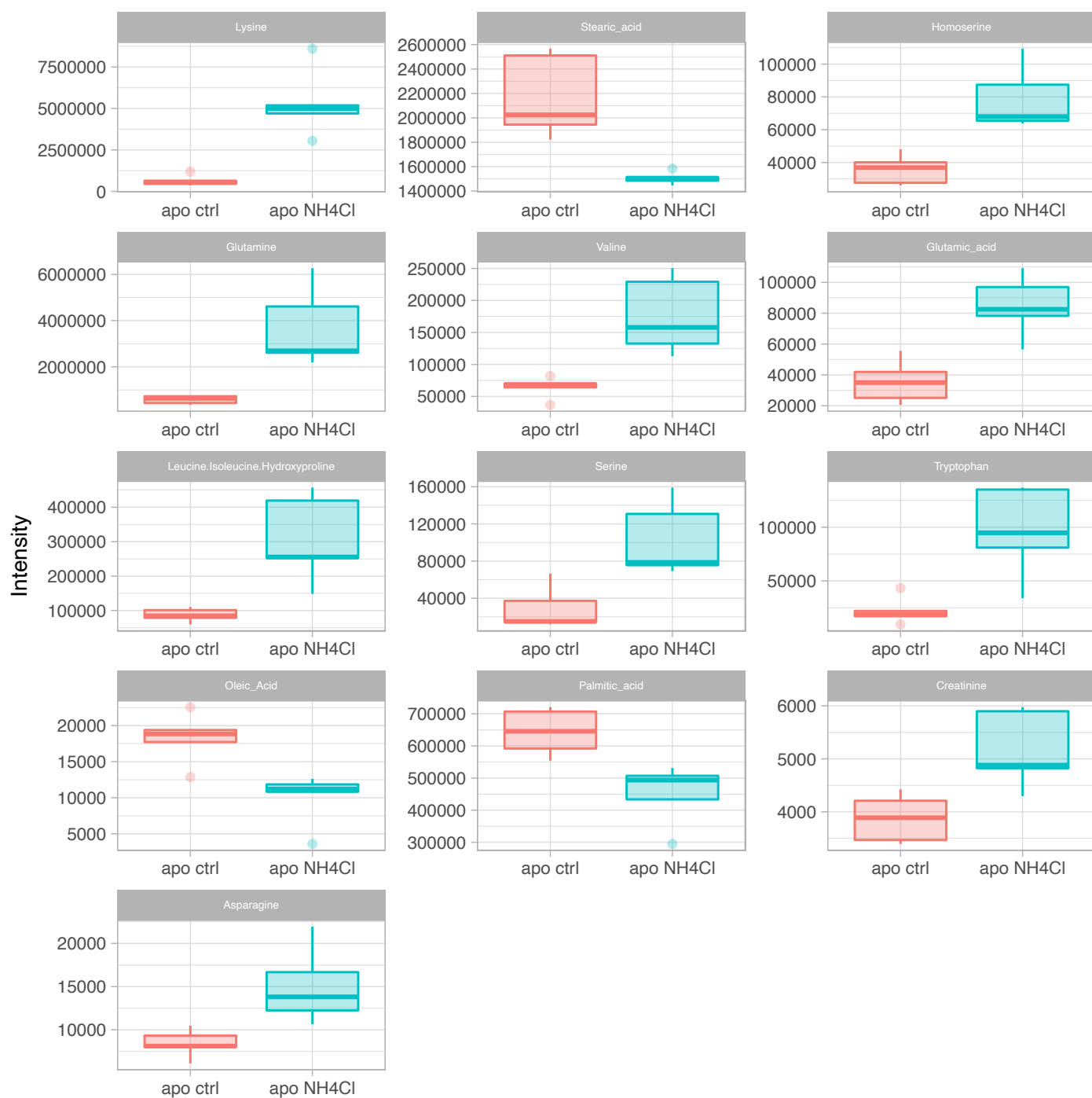

Figure S3

Supplement: Supplementary file 4 — Supplementary Figure 3. [file 41598_2024_53294_MOESM4_ESM.pdf]

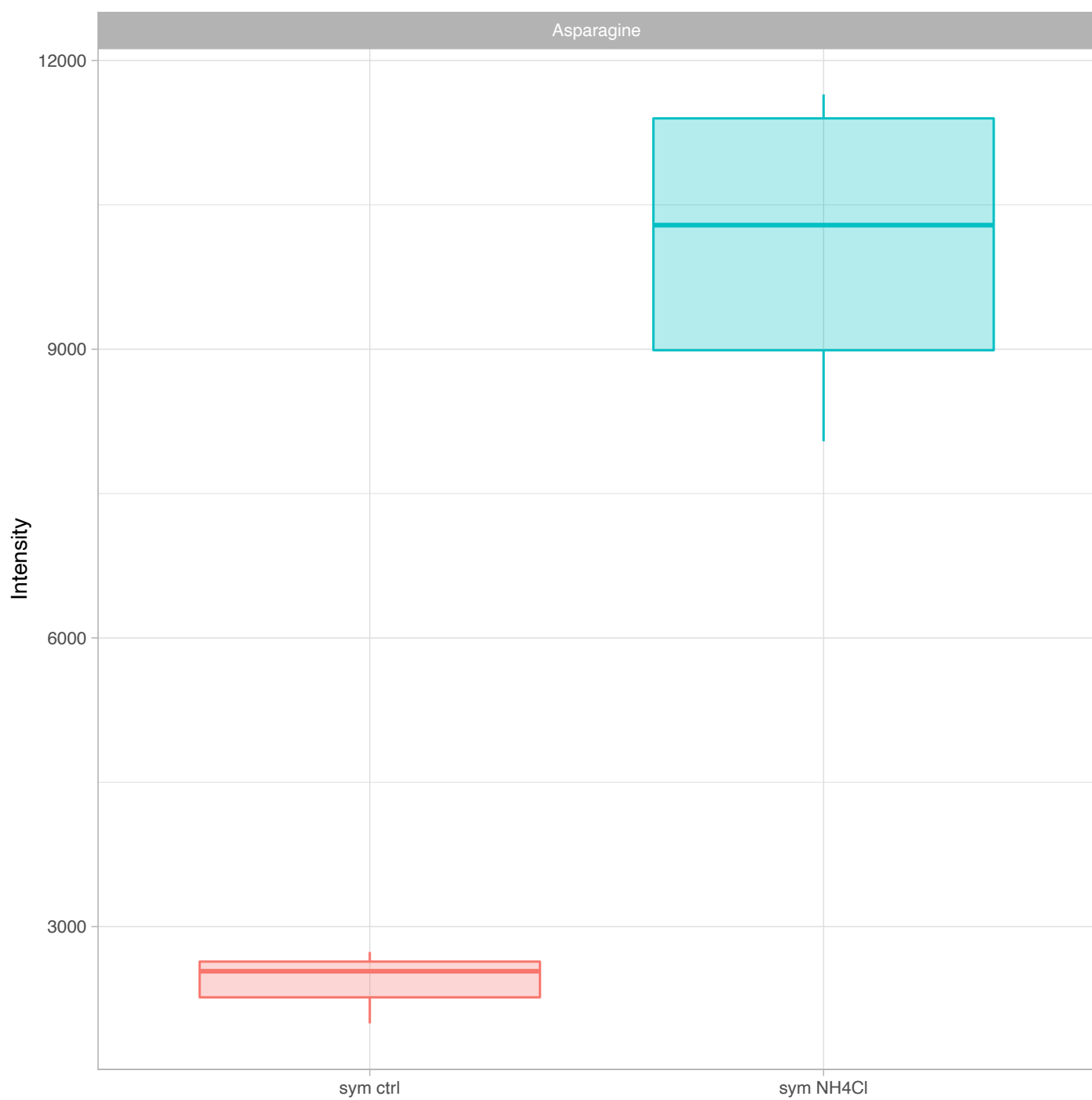

Figure S4

Supplement: Supplementary file 5 — Supplementary Figure 4. [file 41598_2024_53294_MOESM5_ESM.pdf]
