# Supplementary material for: Single-polyp metabolomics for coral health assessment
Source: Sci Rep. 2024 Mar 5;14:3369. doi: 10.1038/s41598-024-53294-8 (PMC10914721; doi:10.1038/s41598-024-53294-8)
Supplement: Supplementary file 3 — Supplementary Figure 2. [file 41598_2024_53294_MOESM3_ESM.pdf]

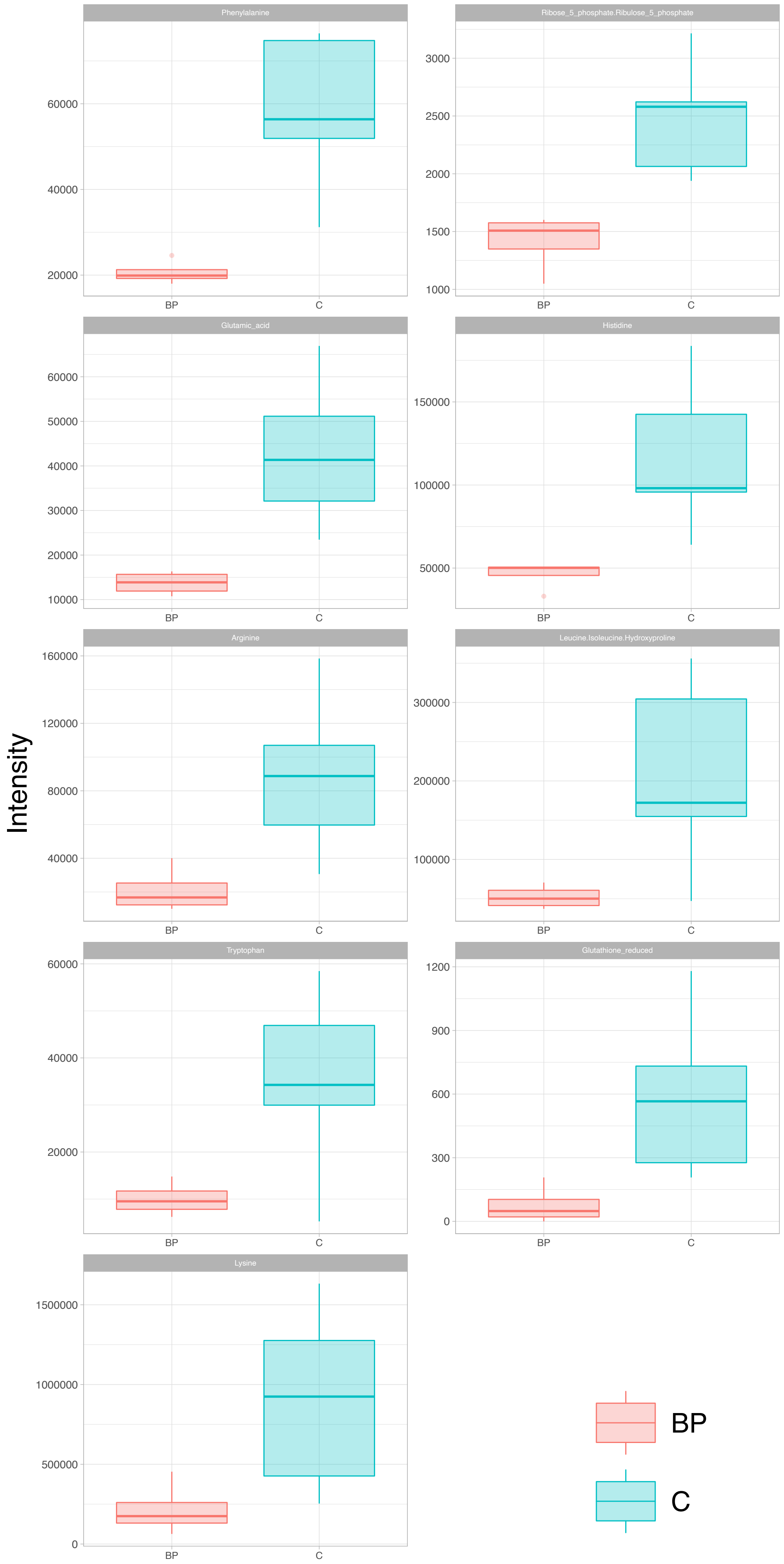
Intensity
Phenylalanine
20000
40000
60000
Ribose_5_phosphate.Ribulose_5_phosphate
1000
1500
2000
2500
3000
Glutamic_acid
10000
20000
30000
40000
50000
60000
Histidine
50000
100000
150000
Arginine
40000
80000
120000
160000
Leucine.Isoleucine.Hydroxyproline
100000
200000
300000
Tryptophan
20000
40000
60000
Glutathione_reduced
0
300
600
900
1200
Lysine
0
500000
1000000
1500000
BP
C
BP
C

Figure S2
